# A novel charged trinuclear platinum complex effective against cisplatin-resistant tumours: hypersensitivity of p53-mutant human tumour xenografts

**DOI:** 10.1038/sj.bjc.6690620

**Published:** 1999-08

**Authors:** G Pratesi, P Perego, D Polizzi, S C Righetti, R Supino, C Caserini, C Manzotti, F C Giuliani, G Pezzoni, S Tognella, S Spinelli, N Farrell, F Zunino

**Affiliations:** Istituto Nazionale per lo Studio e la Cura dei Tumori, Via Venezian 1, Milan, 20133, Italy; Boehringer Mannheim Italia S.p.A., Via G.B. Stucchi 110, Milan, Monza 20052, Italy; Department of Chemistry and Vermont Cancer Center, University of Vermont, Burlington, VT 05405, USA

**Keywords:** multinuclear platinum complex, cisplatin, drug resistance, p53 mutation

## Abstract

Multinuclear platinum compounds were rationally designed to bind to DNA in a different manner from that of cisplatin and its mononuclear analogues. A triplatinum compound of the series (BBR 3464) was selected for preclinical development, since, in spite of its charged nature, it was very potent as cytotoxic agent and effective against cisplatin-resistant tumour cells. Anti-tumour efficacy studies were performed in a panel of human tumour xenografts refractory or poorly responsive to cisplatin. The novel platinum compound exhibited efficacy in all tested tumours and an impressive efficacy (including complete tumour regressions) was displayed in two lung carcinoma models, CaLu-3 and POCS. Surprisingly, BBR 3464 showed a superior activity against p53-mutant tumours as compared to those carrying the wild-type gene. The involvement of p53 in tumour response was investigated in an osteosarcoma cell line, SAOS, which is null for p53 and is highly sensitive to BBR 3464, and in the same cells following introduction of the wild-type p53 gene. Thus the pattern of cellular response was investigated in a panel of human tumour cells with a different p53 gene status. The results showed that the transfer of functional p53 resulted in a marked (tenfold) reduction of cellular chemosensitivity to the multinuclear platinum complex but in a moderate sensitization to cisplatin. In addition, in contrast to cisplatin, the triplatinum complex was very effective as an inducer of apoptosis in a lung carcinoma cell line carrying mutant p53. The peculiar pattern of anti-tumour activity of the triplatinum complex and its ability to induce p53-independent cell death may have relevant pharmacological implications, since p53, a critical protein involved in DNA repair and induction of apoptosis by conventional DNA-damaging agents, is defective in several human tumours. We suggest that the peculiar DNA binding properties of the triplatinum complex may contribute to the striking profile of anti-tumour efficacy. Taken together, the available information supports that anti-tumour activity of the novel compound is mediated by a mechanism different from that of conventional platinum complexes, and compounds of this series could represent a new class of promising anti-tumour agents. © 1999 Cancer Research Campaign


*British Journal of Cancer* (1999) **80**(12), 1912–1919
© 1999 Cancer Research Campaign
Article no. bjoc.1999.0620

### A novel charged trinuclear platinum complex effective against cisplatin-resistant tumours: hypersensitivity of p53-mutant human tumour xenografts

**G Pratesi[1], P Perego[1], D Polizzi[1], SC Righetti[1], R Supino[1], C Caserini[1], C Manzotti[2], FC Giuliani[2], G Pezzoni[2], S Tognella[2], S Spinelli[2], N Farrell[3] and F Zunino[1]**

[1]Istituto Nazionale per lo Studio e la Cura dei Tumori, Via Venezian 1, 20133 Milan, Italy; [2]Boehringer Mannheim Italia S.p.A., Via G.B. Stucchi 110, 20052 Monza (Milan), Italy; [3]Department of Chemistry and Vermont Cancer Center, University of Vermont, Burlington, VT 05405, USA

**Summary** Multinuclear platinum compounds were rationally designed to bind to DNA in a different manner from that of cisplatin and its mononuclear analogues. A triplatinum compound of the series (BBR 3464) was selected for preclinical development, since, in spite of its charged nature, it was very potent as cytotoxic agent and effective against cisplatin-resistant tumour cells. Anti-tumour efficacy studies were performed in a panel of human tumour xenografts refractory or poorly responsive to cisplatin. The novel platinum compound exhibited efficacy in all tested tumours and an impressive efficacy (including complete tumour regressions) was displayed in two lung carcinoma models, CaLu-3 and POCS. Surprisingly, BBR 3464 showed a superior activity against p53-mutant tumours as compared to those carrying the wild-type gene. The involvement of p53 in tumour response was investigated in an osteosarcoma cell line, SAOS, which is null for p53 and is highly sensitive to BBR 3464, and in the same cells following introduction of the wild-type p53 gene. Thus the pattern of cellular response was investigated in a panel of human tumour cells with a different p53 gene status. The results showed that the transfer of functional p53 resulted in a marked (tenfold) reduction of cellular chemosensitivity to the multinuclear platinum complex but in a moderate sensitization to cisplatin. In addition, in contrast to cisplatin, the triplatinum complex was very effective as an inducer of apoptosis in a lung carcinoma cell line carrying mutant p53. The peculiar pattern of anti-tumour activity of the triplatinum complex and its ability to induce p53-independent cell death may have relevant pharmacological implications, since p53, a critical protein involved in DNA repair and induction of apoptosis by conventional DNA-damaging agents, is defective in several human tumours. We suggest that the peculiar DNA binding properties of the triplatinum complex may contribute to the striking profile of anti-tumour efficacy. Taken together, the available information supports that anti-tumour activity of the novel compound is mediated by a mechanism different from that of conventional platinum complexes, and compounds of this series could represent a new class of promising anti-tumour agents.

**Keywords:** multinuclear platinum complex; cisplatin; drug resistance; p53 mutation

Cisplatin is one of the most effective drugs available for the treatment of a number of human solid tumours (in particular, testicular and ovarian carcinoma). The clinical success of cisplatin has stimulated intensive effort in the development of new platinum compounds with improved pharmacological properties. In spite of such effort, only carboplatin has emerged for clinical use as an alternative analogue of cisplatin with comparable efficacy but an improved toxicity profile. Unfortunately, development of drug resistance is a major obstacle to successful treatment of human tumours with cisplatin or carboplatin. The molecular basis of resistance to cisplatin and related mononuclear analogues has not been conclusively defined. Various mechanisms have been implicated, including a number of cellular factors that affect the level of drug reaching DNA and an enhanced DNA repair or increased tolerance to DNA damage (Chu, 1994; Kelland, 1994; Fink et al, 1996; Crul et al, 1997). Although the proposed mechanisms may contribute to the development of a variable degree of cellular resistance, none of them completely explains the intrinsic resistance of several tumours or the pattern of cross-resistance among different DNA-damaging agents.

*Received 4 September 1998*
*Revised 12 February 1999*
*Accepted 23 February 1999*

*Correspondence to:* F Zunino

Conventional platinum compounds (cisplatin and carboplatin) are believed to mediate their cytotoxic effects through their interaction with DNA and formation of a variety of DNA adducts (Eastman, 1987). Among the new platinum compounds with potential activity against cisplatin-resistant cells are multinuclear platinum complexes (Farrell, 1996). Such compounds, containing two reactive platinum centers stably linked by a variable length alkanediamine chain, were originally designed to form different types of DNA adducts, such as ‘long-distance’ intra- and interstrand cross-links not available to conventional (i.e. mononuclear) platinum complexes (Farrell, 1995*a*, 1995*b*). The structural features and the multifunctional nature of such compounds are expected to enhance the sequence specificity at DNA binding sites (Farrell, 1995*b*). By structure–activity relationship studies, we selected for further development a trinuclear platinum complex (BBR 3464) characterized by a promising profile of cytotoxic activity and efficacy against cisplatin-resistant cell lines (Farrell, 1997). The chemical structure and conformation of BBR 3464 are shown in Figure 1.

**A**

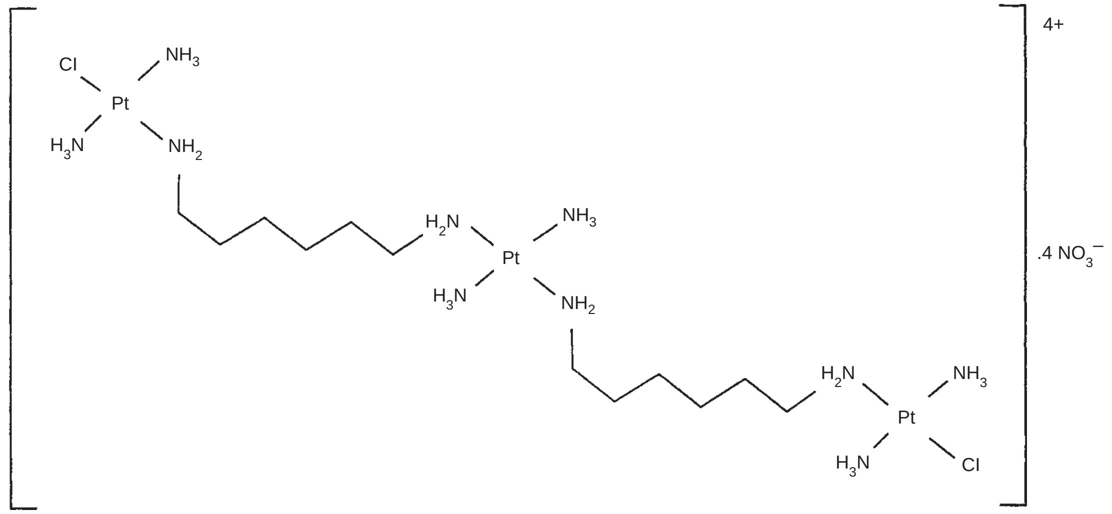

**B**

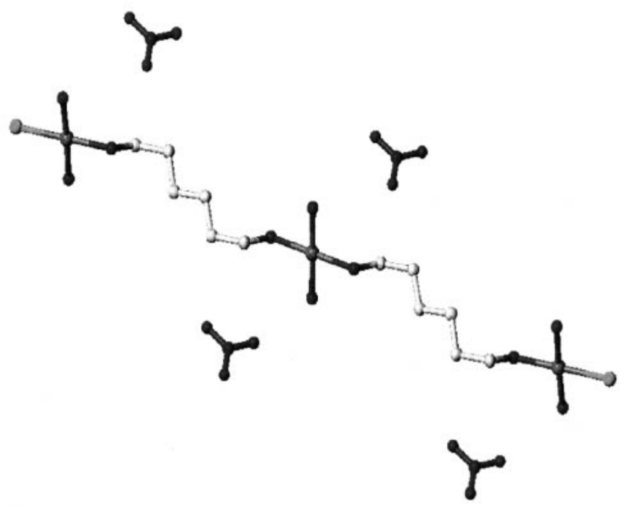

**Figure 1** Chemical structure (**A**) and conformation of BBR 3464 (**B**)

We explored the anti-tumour effects of BBR 3464 in a large number of cisplatin-resistant solid human tumours, with the aim to define the profile of preclinical anti-tumour efficacy. In this study, we documented an improved efficacy of the novel complex in all tested models and a marked efficacy in carcinoma models with mutant p53. A role of wild-type p53 in determining resistance to the novel platinum compound was also supported by cellular pharmacology studies.

### MATERIALS AND METHODS

### Drugs

BBR 3464 was prepared as $NO_3$ salt (Figure 1A) (Farrell, 1997). The structure identity was confirmed by two independent synthetic methods and further supported by X-ray crystallography data (Farrell et al, manuscript in preparation) (Figure 1B). Cisplatin was a product of Bristol-Myers Squibb. All drugs were dissolved in saline immediately before use.

### In vivo studies

Human tumour lines were derived from cell cultures (CaLu-3, POVD/DDP, A2780/CP, IGROV/DDP, DU145, H460, A549, SKOV-3) or directly from surgical specimens (LX-1, POCS). They were maintained in athymic nude mice by serial subcutaneous (s.c.) passages of tumour fragments. Athymic Swiss nude mice, 10–12 weeks old, were used throughout the study (Charles River Laboratories, Calco, Italy). Mice were maintained in laminar flow-rooms, according to the guidelines of the Istituto Nazionale per lo Studio e la Cura dei Tumori of Milan. Experimental protocols were approved by the Ethics Committee for Animal Experimentation according to the UKCCCR guidelines (Workman et al, 1988).

For chemotherapy experiments, mice were s.c. grafted with tumour fragments in both flanks. Each experimental group consisted of 8–10 tumours. Tumour growth was followed and tumour weight (TW) was calculated measuring tumour diameters with a vernier caliper and using the formula: TW (mg) = tumour volume ($mm^3$) = $d^2 \times D/2$, where d and D represent the shortest and the longest diameter respectively.

When tumours weighed around 50–100 mg, mice were treated intravenously with cisplatin or BBR 3464 (both dissolved in saline). Treatments were repeated weekly for a total of 3 times (q7d×3). Drug efficacy was assessed using two conventional parameters:

1. relative tumour weight inhibition (RTWI%) in treated (T) versus control (C) mice according to the formula: $100-[TW_T/TW_c \times 100]$
2. specific growth delay (SGD) calculated as $(T-C)/C$ where T is days for treated tumours and C days for control tumours taken to double (SGD 1–2) or quadruplicate (SGD 1–4) their initial weight.

A tumour was considered responsive when the treatment achieved an SGD ≥ 1.5. Mean values were used for all calculations. Mice were weighed weekly during the experimental time frame, and results are presented for drug doses causing a body weight loss ≤ 15%.

Statistical comparison of TW in cisplatin- vs BBR 3464-treated tumours was assessed by Student's *t*-test (two-tailed).

*© 1999 Cancer Research Campaign* 

**Table 1** p53 status in human tumour models

| Tumour model | Tumour type | Resistance | p53 status[a] Mutation Exon | Codon | Nucleotide change | Amino acids substitution |
|---|---|---|---|---|---|---|
| p53 wild-type | | | | | | |
| H460 | non-SCLC | Intrinsic | | | | |
| A549 | non-SCLC | Intrinsic | | | | |
| A2780/CP | Ovarian | Acquired | | | | |
| SKOV-3[b] | Ovarian | Intrinsic | | | | |
| p53 mutant | | | | | | |
| CaLu-3 | non-SCLC | Intrinsic | 7 | 237 | ATG→ATT (L) | Met→Ile |
| LX-1 | non-SCLC | Intrinsic | 9 | 309 | CCC→TCC | Pro→Ser |
| POCS | SCLC | Intrinsic | 7 | 242 | TGC→TTC (L) | Cys→Phe |
| POVD/DDP | SCLC | Acquired | 5 | 171 | GAG→TAG (LH) | Glu→Stop |
| IGROV/DDP | Ovarian | Acquired | 8 | 270 | TTT→TTA | Phe→Leu |
| | | | | 282 | CGG→TGG (LSH) | Arg→Trp |
| DU145 | Prostatic | Intrinsic | 6 | 223 | CCT→CTT | Pro→Leu |
| | | | 8 | 274 | GTT→TTT (LSH) | Val→Phe |

[a]Exons 5–9 were analysed. In parenthesis: structural/functional domain of p53 according to Volgestein and Kinzler (1994). [b]No endogenous expression of p53. SCLC, small-cell lung carcinoma.

**Table 2** Comparison of efficacy of cisplatin and BBR 3464 on human carcinoma xenografts (s.c.). Treatment i.v., q7dx3

| Tumour model | Growth rate[a] 1–2 | Growth rate[a] 1–4 | Drug | Optimal dose[b] ($mg\ kg^{-1}$) | TWI[c] (%) | SGD[d] 1–2 | SGD[d] 1–4 |
|---|---|---|---|---|---|---|---|
| H460 | 2.5 | 7.5 | Cisplatin | 6 | 42 | 0.4 | 0.6 |
| | | | BBR 3464 | 0.4 | 64** | 0.6 | 1.6 |
| A549 | 7.5 | 19.5 | Cisplatin | 6 | 18 | 0 | 0.5 |
| | | | BBR3464 | 0.3 | 45* | 0 | 1.6 |
| | | | | 0.4 | 64* | 2.3 | 3.1 |
| A2780/CP | 2.5 | 5 | Cisplatin | 4 | 48 | 0.2 | 0.2 |
| | | | BBR 3464 | 0.3 | 63 | 0.2 | 2.0 |
| SKOV-3 | 5.5 | 13 | Cisplatin | 6 | 21 | 0.4 | 0.3 |
| | | | BBR 3464 | 0.4 | 60* | 1.9 | 2.4 |
| CaLu-3 | 5.5 | 11 | Cisplatin | 6 | 60 | 1.2 | 0.9 |
| | | | BBR 3464 | 0.3 | 92***(5/11) | ND | ND |
| LX-1 | 3.7 | 7.5 | Cisplatin | 4 | 39 | 0.4 | 0.5 |
| | | | BBR 3464 | 0.3 | 77* | 0.8 | 2.2 |
| POCS | 12 | 21 | Cisplatin | 6 | 56 | 0.1 | 0.75 |
| | | | BBR 3464 | 0.4 | 92**(4/6) | ND | ND |
| POVD/DDP | 3.5 | 7 | Cisplatin | 6 | 70 | 0.7 | 1.0 |
| | | | BBR 3464 | 0.3 | 85* | 1.6 | 3.3 |
| | | | | 0.4 | 93** | 8.1 | 4.9 |
| IGROV/DDP | 4 | 8.5 | Cisplatin | 6 | 68 | 0.75 | 1.5 |
| | | | BBR 3464 | 0.3 | 80 | 3.25 | 3.7 |
| DU145 | 3 | 8 | Cisplatin | 6 | 30 | 0.2 | 0.4 |
| | | | BBR 3464 | 0.4 | 63 | 2.0 | 1.4 |

[a]Days required for control tumours to double or quadruplicate their initial weight. [b]Maximum tolerated dose with the indicated schedule. [c]Inhibition of relative tumour weight in treated versus control mice. *$P < 0.05$; **$P < 0.01$; ***$P < < 0.01$ (by Student's *t*-test) versus cisplatin-treated tumours in the same experiment. In parenthesis, partial or complete tumour regressions at the end of experiment. [d]Specific growth delay in treated compared to control tumours to reach twice (1–2) and four times (1–4) their initial weight. Calculations were done as specified in the Methods. ND, not determined because of partial or complete regression of treated tumours.

### Cell lines and cytotoxicity studies

Tumour cell lines were maintained in RPMI-1640 plus 10% fetal calf serum. Cytotoxicity was assessed by growth-inhibition assay. Briefly, 24 h after seeding, cells were exposed to the drug for 1 h and then incubated in drug-free medium for 72 h and counted with a ZBI Coulter counter (Coulter Electronics Ltd, Luton, UK). In long-term exposures, cells were maintained in the presence of drug for 72 h. The 50% inhibitory concentration ($IC_{50}$) was calculated from dose–response curves.

### Apoptosis and cell cycle analysis

The extent of apoptosis was assessed by fluorescence microscopy as already described (Perego et al, 1996; Caserini et al, 1997). Briefly, at the end of exposure to drug, cells were fixed in cold

 *© 1999 Cancer Research Campaign*

**Table 3** Cytotoxic effects of cisplatin and BBR 3464 in human tumour cell lines with different p53 status or expression

| Cell lines (tumour type) | p53 status | IC$_{50}$ (µg/ml)[a] Cisplatin 1 h | Cisplatin 72 h | BBR 3464 1 h | BBR 3464 72 h |
|---|---|---|---|---|---|
| A2780 (ovarian carcinoma) | Wild-type | 1.2 ± 0.03 | 0.06 ± 0.01 | 0.032 ± 0.001 | 0.012 ± 0.004 |
| A2780/CP (ovarian carcinoma) | Wild-type | 35 ± 2.8 (29) | 0.73 ± 0.2 (12) | 2.0 ± 0.2 (62) | 0.29 ± 0.2 (24) |
| IGROV-1 (ovarian carcinoma) | Wild-type | 4.3 ± 1.3 | | 8.0 ± 2.8 | |
| U2-OS (osteosarcoma) | Wild-type | 2.4 ± 0.1 | | 1.7 ± 0.01 | |
| SW626 (ovarian carcinoma) | Mutant (codon 273) | 6.3 ± 3.0 | | 0.08 ± 0.03 | |
| SAOS (osteosarcoma) | Null | 3.0 ± 0.2 | | 0.1 ± 0.01 | |
| POGB (SCLC) | Mutant (codon 282) | | 0.25 ± 0.07 | | 0.009 ± 0.006 |
| A431 (cervical carcinoma) | Mutant (codon 273) | 10.4 ± 3 | | 3.1 ± 1.8 | |

[a]Cytotoxicity following the indicated exposure to the drug was determined by growth inhibition test as described in the Methods. IC$_{50}$ values (±SD) were deduced from dose–response curves. In parenthesis: resistance index.

70% ethanol, stained with propidium iodide (PI) solution (30 µg ml$^{-1}$ PI and 66 U ml$^{-1}$ RNAase in phosphate-buffered saline) and stored in the dark for 30 min. At least 100 cells in two different smears for each sample were examined for morphological changes. The percentage of apoptotic cells was referred to the total cell number. To investigate cell cycle distribution the fluorescence intensity of PI-stained cells was determined by FACScan flow cytometer (Becton Dickinson, Mountain View, CA, USA).

### Transfection of p53-deficient SAOS cells with a wild-type p53 expression vector

SAOS cells were seeded in 6-well plates and transfected when they reached 70% confluency. Each well was incubated for 4 h with serum-free medium containing 30 µl of lipofectin (Gibco BRL, Life Technologies Inc., Gaithersburg, MD, USA) and 3 µg of DNA consisting of the wild-type p53 expression plasmid pCMV-SN3 or a parental of the former as control. The pCMV-SN3 plasmid was kindly supplied by Dr Bert Vogelstein (Johns Hopkins Oncology Center, Baltimore, MD, USA). Medium was replaced with serum containing medium and 48 h later selection was started by adding 400 µg ml$^{-1}$ geneticin. Geneticin-resistant cells were maintained in selective medium.

### Western blot analysis

Western blot analysis of cell lysates was performed as previously described (Perego et al, 1996). Briefly, samples were fractionated by sodium dodecyl sulphate polyacrylamide gel electrophoresis (SDS-PAGE) and blotted on nitrocellulose sheets. Blots were then preblocked for 1 h at room temperature in phosphate-buffered saline containing 5% (w/v) dried non-fat milk. Filters were incubated overnight at 4°C with an antibody to p53 (DO-7, Dako, Glostrup, Denmark) to p21$^{WAF1}$ (Neomarkers, Fremont, CA, USA) or to MDM2 (Oncogene Research, Cambridge, MA, USA). A rabbit antiserum to recombinant human HAP1 or to actin (Sigma, St Louis, MO, USA) was used as control for loading since these proteins are equally expressed in the studied cell lines. Antibody binding to filters was detected by the enhanced chemiluminescence luciferase method (Amersham, Little Chalfont, UK).

### SSCP and sequencing analysis of the p53 gene

Single-strand conformation polymorphism (SSCP) and sequencing analysis for detection of p53 gene mutations in the most affected exons 5–9 were performed as previously described (Perego et al, 1996). Briefly, SSCP bands recovered from gels were used as templates in direct sequencing reactions using the Amplitaq Cycle Sequencing kit (Perkin-Elmer, Branchburg, NJ, USA). In Tables 1 and 3, p53 gene is referred as wild-type if mutations in exons 5–9 were not detected.

### RESULTS

### Anti-tumour efficacy studies

The biological and molecular profiles of the human tumour models used in this study are summarized in Table 1. The panel included ten carcinoma xenografts of different tumour types, including six lung, three ovarian and one prostatic carcinoma. The tumours were chosen, because, in our experience, they were relatively resistant to cisplatin. The resistant status was more evident for the seven tumours with intrinsic resistance. In all our tumour models, we determined the p53 status. The SSCP and sequence analysis revealed p53 mutations in 6/10 tumours. The pattern of tumour response to cisplatin or to the multinuclear complex BBR 3464 is shown in Table 2, which summarizes the effects of drug treatment using optimal doses for each agent (i.e. maximum

© 1999 Cancer Research Campaign 

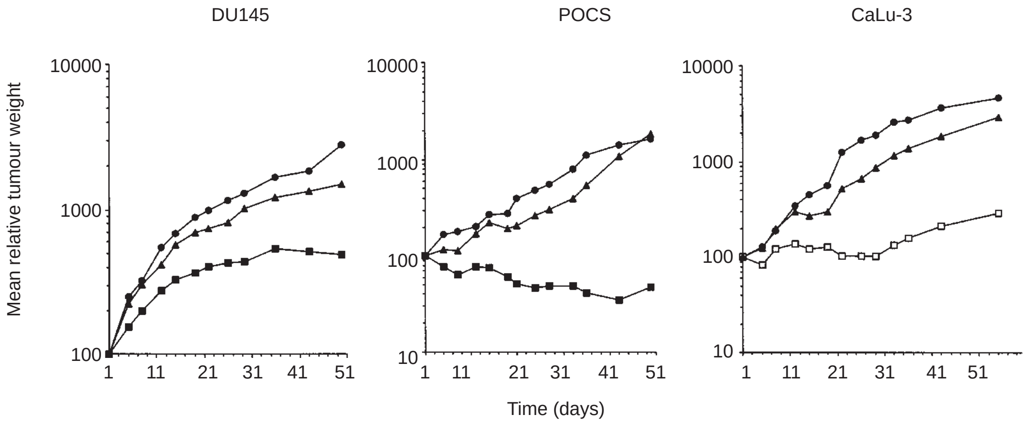

**Figure 2** In vivo response of human tumour xenografts following treatment with cisplatin or BBR 3464. Abscissa: days after first drug treatment. Ordinate: mean relative tumour weights (TW), calculated by dividing the mean TW at each time by the mean TW at day 1 (1st day of treatment) × 100. Drug treatments were delivered every 7 days for 3 times. Untreated controls, •; cisplatin, 6 mg kg$^{-1}$, ▲; BBR 3464, 0.3 mg kg$^{-1}$, □ or 0.4 mg kg$^{-1}$, ■. See Table 2 and Methods for details

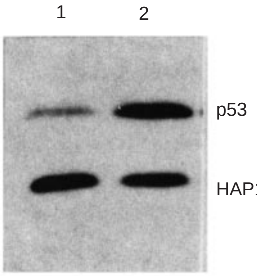

**Figure 3** Western blot analysis of p53 in A2780 (1) and A2780/CP (2) cells. Control loading for protein is shown by HAP1

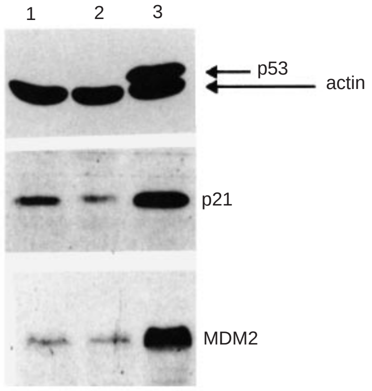

**Figure 4** Western blot analysis of p53, p21 and MDM2 in p53-deficient and wild-type p53-transfected SAOS cells. 1, p53-deficient cells; 2, empty vector transfected cells; 3, wild-type p53-transfected cells. Control loading is shown by actin

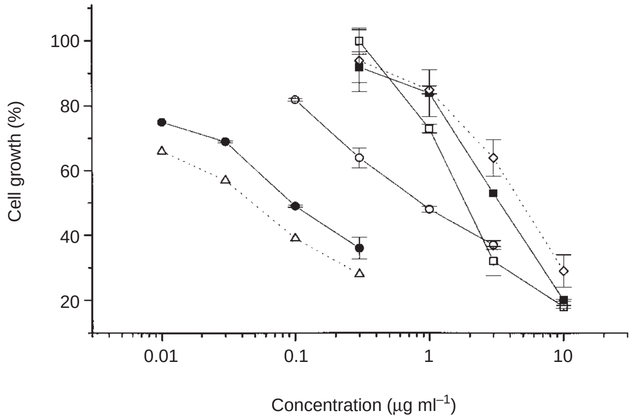

**Figure 5** Effect of transfection of p53-deficient SAOS cells with a wild-type p53 expression vector on cytotoxicity of cisplatin or BBR 3464. Cells were exposed to the drug for 1 h and cytotoxic effect was determined by the growth inhibition test as described in the Methods. (■) SAOS cells treated with cisplatin; (●) BBR 3464-treated SAOS cells; wt-p53 expressing cells treated with cisplatin (□) or BBR 3464 (○); empty vector-transfected cells treated with cisplatin (◇) or BBR 3464 (Δ)

tolerated doses causing ≤ 15% body weight loss and ≤ 10% of lethality). Cisplatin treatment achieved less than 60% TWI in seven of the ten tumour systems. Although in three other tumours drug effects were pharmacologically appreciable (TWI between 60 and 70%), all tumours of this panel should be considered resistant or poorly responsive to cisplatin, as evidenced by marginal SGD values (≤ 1.5). In contrast, BBR 3464 exhibited a remarkable antitumour activity, resulting in TWI > 60% and SGD > 1.5, in all tested tumour models. In most tumour systems, the inhibition lasted for a long time, as documented by SGD values (Table 2) and shown in representative growth curves (Figure 2). A striking finding of this study was hypersensitivity of most p53-mutant tumours (TWI > 75% in five tumours) compared to tumours with the wild-type gene. In the tumours carrying wild-type p53, TWI achieved by BBR 3464 did not exceed 75%. Among the p53-mutant tumours, impressive was the activity against three lung carcinomas, i.e. CaLu-3 and POCS, in which partial or complete regressions were produced in most treated tumours, and POVD/DDP, in which the highest SGD value was achieved. In spite of the presence of wild-type p53 gene, SKOV-3 should be regarded as p53-deficient model, since no protein expression could be detected. Again, this tumour model was markedly more responsive to BBR 3464 than to cisplatin, which was almost ineffective. The activity achieved by BBR 3464 in this tumour is relevant, considering its intrinsic resistance to several conventional agents.

 *© 1999 Cancer Research Campaign*

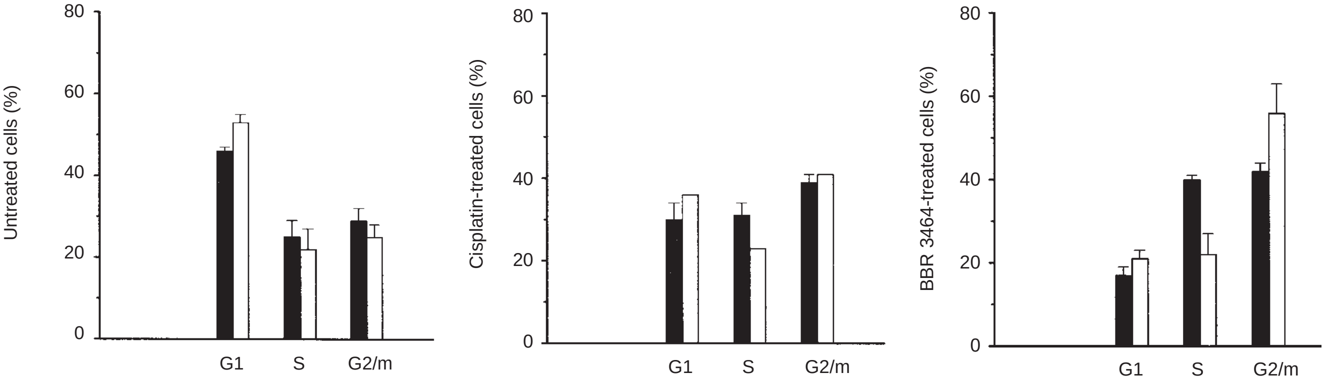

**Figure 6** Cell cycle distribution of p53-deficient (■) and wild-type p53-transfected SAOS cells (□) exposed to cisplatin or BBR 3464. Seventy-two hours after a 1 h exposure to the drug (3 μg $ml^{-1}$), cells were processed for FACScan analysis. The percentage of cells in the different phases of cell cycle was obtained by computer analysis of DNA hystograms

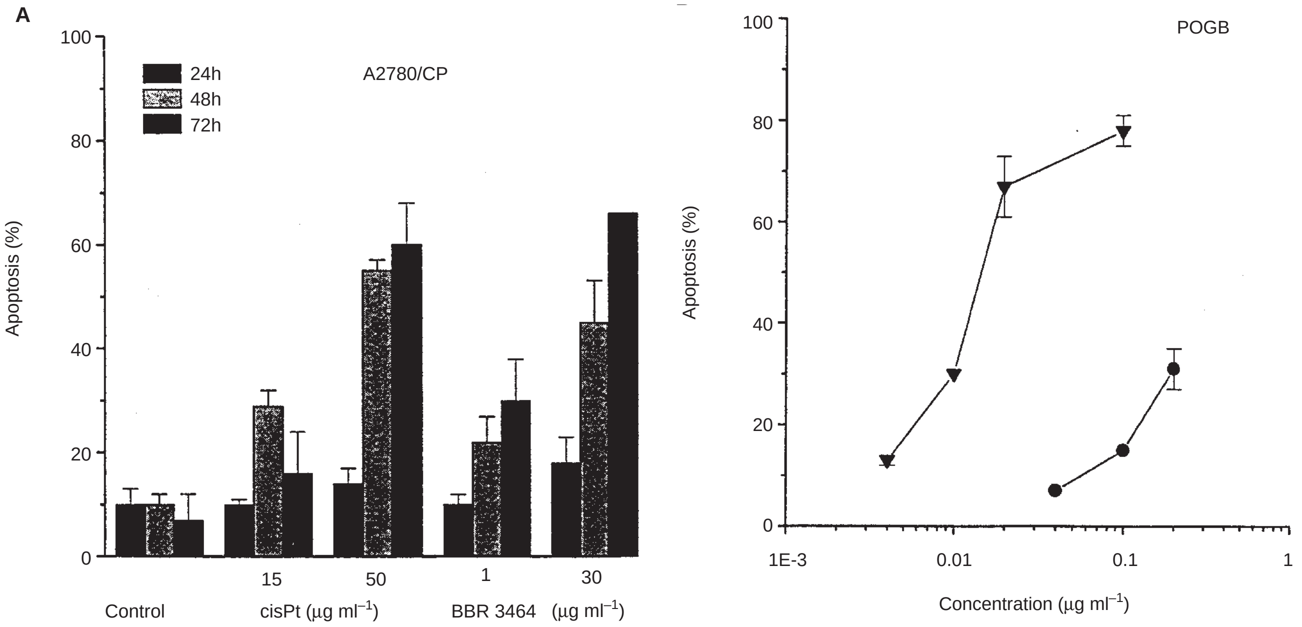

**Figure 7** Apoptosis induction by cisplatin or BBR 3464. (**A**) A2780/CP cells were exposed to the drug for 1 h and the extent of apoptotic cells was determined at the indicated times; (**B**) POGB cells were exposed to cisplatin (●) or BBR 3464 (▲) for 72 h. The percentage of apoptotic cells in the whole population was determined by fluorescence microscopic observation of propidium iodide-stained cells. At least 100 cells from two different smears were examined for nuclear morphological changes

### Pattern of chemosensitivity of human tumour cells

Based on observations concerning anti-tumour activity, a comparative study of cellular response to cisplatin and BBR 3464 was performed in a panel of eight human tumour cell lines of various tumour types. The panel included four cell lines containing wild-type p53 and four p53-deficient cell lines. The pattern of cellular response (Table 3) was consistent with in vivo tumour response, since in general p53-mutant or deficient cells were more sensitive to BBR 3464 than cell lines with wild-type p53. In contrast, p53-mutant cells showed comparable or somewhat reduced sensitivity to cisplatin compared to wild-type p53-containing cells. Among tumour cells with wild-type p53, the cisplatin-resistant subline (A2780/CP) exhibited a marked cross-resistance to BBR 3464. The observation that an increased expression of p53 protein was present in the resistant subline (Figure 3) is consistent with a role of p53 in cellular response to BBR 3464. The functionality of p53 in A2780/CP is questionable, since no elevation of $p21^{WAF-1}$ protein could be detected after exposure to cisplatin (Perego et al, 1997). However, based on the multifunctional nature of p53, an involvement of p53 could not be ruled out, as suggested by an increased cell ability to repair DNA damage (Caserini et al, 1997).

No precise correlation between p53 status and drug sensitivity can be expected from our panel of cell lines, since multiple factors affecting chemosensitivity may be expressed in a variable manner in cells of different tumour types. Thus, to overcome the difficulty in separating the effects of multiple alterations expected in non-isogenic systems, the role of p53 in response to platinum compounds was investigated in a comparative study of osteosarcoma cells, SAOS (defective in p53 function), following transfection with the wild-type gene. A polyclonal population expressing p53, and two p53-regulated genes ($p21^{WAF1}$ and MDM2) was used

*© 1999 Cancer Research Campaign* 

(Figure 4). Introduction of a functional p53 gene significantly reduced (around tenfold) sensitivity of SAOS cells to BBR 3464 (Figure 5). An opposite effect was found on cisplatin sensitivity, since transfection resulted in an appreciable sensitization. The cell cycle analysis indicated that cisplatin induced a comparable perturbation of cell cycle in the isogenic systems, as expected for a DNA damaging agent (i.e. accumulation of cells in G2 phase). This effect was also evident in BBR 3464-treated cells but more marked in p53-tranfected cells, thus suggesting that the role of p53 in G2-checkpoint may be more relevant for DNA lesions induced by the multinuclear platinum complex (Figure 6).

Since cellular sensitivity to platinum compounds has been related to their ability to induce apoptosis, drug ability to trigger an apoptotic response was explored in two cell lines of our panel, including the resistant ovarian carcinoma A2780/CP, with wild-type p53 gene sequence and the small-cell lung carcinoma cell line POGB, with mutant gene (Supino et al, 1996). In both cell systems, the increased cytotoxic potency of BBR 3464, compared to cisplatin, reflected a marked susceptibility to apoptosis induction (Figure 7).

### DISCUSSION

This study showed that the cationic trinuclear platinum complex, BBR 3464, is characterized by a marked increase (up to 50-fold) in cytotoxic potency as compared to cisplatin and an outstanding efficacy in the treatment of cisplatin-resistant tumours. The increased cytotoxic and anti-tumour potency is somewhat surprising on the basis of its charged nature. The high level of anti-tumour activity in all cisplatin-resistant tumours suggests the drug ability to overcome multiple mechanisms of cisplatin resistance. Among the tumours included in our panel, the BBR 3464 complex exhibited an impressive level of anti-tumour activity against tumours containing a mutant p53. The pattern of tumour response was surprising, since loss of wild-type p53 function could lead to intrinsic resistance as a consequence of a reduced susceptibility to apoptosis following DNA damage. Indeed, apoptosis is recognized as a major mode of cell death induced by cisplatin as well as many DNA-damaging anti-tumour agents (Perego et al, 1996; Zunino et al, 1997).

Since in all p53-mutant tumours no wild-type sequence could be detected at the site of mutation, a consistent loss of function of wild-type p53 was expected. In particular, the mutation found in CaLu-3, resulting in substitution of Met with Ile at the start of loop 3 of p53 (residue 237) has been shown to give a completely mutant phenotype in terms of conformational analysis (Rolley et al, 1995). The mutation found in LX-1 (codon 309), localized outside the central evolutionary conserved sequence-specific DNA binding domain, involves a phosphorylation site required alone or with $Ser_{313}$ for p53 DNA binding activity (Hecker et al, 1996). POCS exhibits a mutation (codon 242) which behaves as dominant negative, as documented by assays carried out in yeast (Brachmann et al, 1996). Based on the crystal structure of the central portion of human p53 complexed with DNA (Vogelstein et al, 1994), such a mutation is conceivable to destabilize p53 altering its functionality, since $Cys^{242}$ participates in coordinating a Zn atom to the protein loops. Regarding the POVD/DDP tumour, the codon 171 mutation is expected to lead to a truncated protein lacking most of the regions required for p53 oligomerization (319–360) and for DNA damage recognition (311–393). Loss of p53 function has been observed in the cellular system from which IGROV/DDP xenograft was originated. These cells, carrying p53 mutation at codons 270 and 282, lack G1 control after radiation and p21 induction after DNA damage (Perego et al, 1996). At least one of the two mutations found in DU145 (codon 223) has been previously shown to result in loss of p53 functionality, as indicated by loss of G1 arrest, of inducibility of $p21^{WAF1}$ and GADD45 and MDM2 mRNA after radiation (O'Connor et al, 1997). In the p53-mutant tumours of our panel, resistance to cisplatin could be predicted on the basis of recent advances in knowledge of the molecular pathways of apoptosis activated by DNA-damaging agents (Lowe et al, 1994; Hickman, 1996; Zunino et al, 1997). Recently, a correlation has been found between mis-sense mutations and clinical resistance of advanced ovarian carcinoma treated with cisplatin-based therapy (Righetti et al, 1996).

In addition to a role of p53 in modulating apoptosis, p53-mediated cellular responses include cell growth arrest, which is required to allow DNA repair prior to genome replication (Kastan et al, 1995; Zunino et al, 1997). The p53 protein itself has been implicated in the recognition and repair of damaged DNA (Wang et al, 1995; Harris, 1996). Based on p53 functions, a plausible explanation for the hypersensitivity of human tumours with mutant p53 to BBR 3464 is that apoptosis induced by the drug is not mediated by p53, which in the wild-type form could enhance the cell's efficiency to repair DNA damage. If this interpretation is correct, overexpression of wild-type p53 might reduce sensitivity to BBR 3464. Similarly, introduction of wild-type p53 in cells lacking p53 function resulted in a significant resistance to BBR 3464. It is thus conceivable that, in tumour cells containing a functional p53, repair of BBR 3464-induced DNA lesions prevails over apoptosis triggering.

The efficacy of BBR 3464 in tumours with mutant p53 suggests that a p53-independent response can be activated by the DNA lesions induced by this agent. Indeed, a human small-cell lung carcinoma cell line (POGB), characterized by loss of p53 function as a consequence of mutation and relatively resistant to cisplatin (Supino et al, 1996), was very sensitive to BBR 3464, and cytotoxicity reflected a marked susceptibility to apoptosis induction by BBR 3464. An alternative explanation for BBR 3464 efficacy against p53-mutant tumour cells could be a persistent cytostatic effect, likely related to the cell's inability to recognize and repair specific DNA lesions induced by BBR 3464 and resulting in delayed apoptosis or in a different cell death pathway. Such an interpretation is consistent with the persistent tumour growth inhibition observed after in vivo treatment.

The impetus for development of new platinum complexes, with genuinely new structures compared to cisplatin, comes from the consideration that clinical trials of conventional cisplatin analogues have, in general, not produced drugs with an enhanced spectrum of anti-tumour activity. Sequence specificity, conformational changes and interactions with DNA-processing proteins of direct cisplatin analogues are similar to those of the parent drug. Thus, the biological effects are expected to be the same, and differences in anti-tumour activity and toxicity may be dictated by differences in pharmacokinetics or reactivity rather than enhanced inhibition of target (DNA) functions. As a corollary, platinum compounds structurally dissimilar to cisplatin may, by virtue of formation of different types of platinum-DNA adducts, exhibit a spectrum of clinical activity complementary to the parent drug, and it is this hypothesis that has driven our drug design efforts (Farrell, 1993). The bifunctional DNA binding of BBR 3464 is similar to that extensively outlined for dinuclear platinum

 © 1999 Cancer Research Campaign complexes (Farrell, 1996). The central hydrogen-bonding core of BBR 3464 enhances kinetics of binding, but conformational changes of DNA are similar to those previously described. They include a high proportion of interstrand cross-links, where the platinated bases on each strand are separated by up to as many as 4 base pairs, and the irreversible induction of the left-handed Z-DNA conformation (Johnson et al, 1992; Wu et al, 1996). These DNA adducts are unique to the class of di- and trinuclear platinum complexes and are discrete both from cisplatin and the alkylating agents such as melphalan and chloroambucil. The lack of cross-resistance and the increased cytotoxic potency of the novel multi-nuclear platinum complex support a different mechanism of DNA interaction (Perego et al, 1999).

In conclusion, the identification of a new class of DNA-damaging agents exhibiting high activity in p53-mutant tumours was an unpredictable outcome from our programme of systematic synthesis. Further studies could be of fundamental importance in designing even newer agents capable of triggering p53-independent cell death. Indeed, the discovery of agents with preferential toxicity against p53 mutant tumour cells should be considered an important goal in the development of new effective anti-tumour agents (O'Connor et al, 1997), because inactivation of p53 function, as a consequence of mutation or deletion of the gene, occurs in many human tumours, including tumour types relatively responsive to cisplatin (e.g. lung and ovarian carcinoma).

### ACKNOWLEDGEMENTS

This work was supported in part by the American Cancer Society grant to N Farrell and by fellowships to SC Righetti from the Associazione Italiana per la Ricerca sul Cancro and to P Perego from the Ministero della Sanita'. We thank Ms L Zanesi for editorial assistance and Ms N Carenini and Ms M Tortoreto for technical assistance.

### REFERENCES

Brachmann RK, Vidal M and Boeke JD (1996) Dominant-negative p53 mutations selected in yeast hit cancer hot spots. *Proc Natl Acad Sci USA* **93**: 4091–4095

Caserini C, Pratesi G, Tortoreto M, Bedogné B, Carenini N, Supino R, Perego P, Righetti SC and Zunino F (1997) Apoptosis as a determinant of tumour sensitivity to topotecan in human ovarian tumours: preclinical in vitro/in vivo studies. *Clin Cancer Res* **3**: 955–961

Chu G (1994) Cellular responses to cisplatin. *J Biol Chem* **269**: 787–790

Crul M, Schellens JHM, Beijnen JH and Maliepaard M (1997) Cisplatin resistance and DNA repair. *Cancer Treat Rev* **23**: 341–366

Eastman A (1987) The information, isolation and characterization of DNA adducts produced by anticancer platinum complexes. *Pharmacol Ther* **34**: 155–166

Farrell N (1993) Nonclassical platinum antitumour agents: perspective for design and development of new drugs complementary to cisplatin. *Cancer Invest* **11**: 578–589

Farrell N (1995a) DNA binding and chemistry of dinuclear platinum complexes. *Comments in Inorganic Chemistry* **16**: 373–389

Farrell N (1996) DNA binding of dinuclear platinum complexes. In: *Advances in DNA Sequence Specific Agents*, Hurley LH, Chaires JB (eds), pp. 187–216. JAI Press Inc

Farrell N, Appleton TG, Qu Y, Roberts JD, Soares Fontes AP, Skov KA, Wu P and Zou Y (1995b) Effects of geometric isomerism and ligand substitution in bifunctional dinuclear platinum complexes on binding properties and conformational changes in DNA. *Biochemistry* **34**: 15480–15487

Farrell N, Qu Y, Kasparkova J, Brabec V, Valsecchi M, Menta E, Di Domenico R, Conti M, Da Re G, Lotto A and Spinelli S (1997) Chemical studies and DNA binding of charged polynuclear platinum complexes. *Proc Am Ass Cancer Res* **38**: 310

Fink D, Nebel S, Aebi S, Zheng H, Cenni B, Nehmé A, Christen RD and Howell SB (1996) The role of DNA mismatch repair in platinum drug resistance. *Cancer Res* **56**: 4881–4886

Harris CC (1996) Structure and function of the p53 tumour suppressor gene: clues for rational cancer therapeutic strategies. *J Natl Cancer Inst* **88**: 1442–1455

Hecker D, Page G, Lohrum M, Weiland S and Scheidtmann KH (1996) Complex regulation of the DNA-binding activity of p53 by phosphorylation: differential effects of individual phosphorylation sites on the interaction with different binding motifs. *Oncogene* **12**: 953–961

Hickman JA (1996) Apoptosis and chemotherapy resistance. *Eur J Cancer* **32A**: 921–926

Johnson A, Qu Y, Van Houten B and Farrell N (1992) B→Z-DNA conformational changes induced by a family of bis(platinum) complexes. *Nucleic Acids Res* **20**: 1697–1703

Kastan MB, Canman CE and Leonard CJ (1995) P53, cell cycle and apoptosis implications for cancer. *Cancer Met Rev* **14**: 3–15

Kelland LR (1994) The molecular basis of cisplatin sensitivity/resistance. *Eur J Cancer* **30A**: 725–727

Lowe SW, Bodis S, McClatchey A, Remington L, Ruley HE, Fisher DE, Housman DE, Jacks T (1994) p53 status and the efficacy of cancer therapy in vivo. *Science* **266**: 807–810

O'Connor PM, Jackman J, Bae I, Myers TG, Fan S, Mutoh M, Scudiero DA, Monks A, Sausville EA, Weinstein JN, Friend S, Fornace AJ and Kohn KW (1997) Characterization of the *p53* tumour suppressor pathway in cell lines of the National Cancer Institute Anticancer Drug Screen and correlations with the growth-inhibitory potency of 123 anticancer agents. *Cancer Res* **57**: 4285–4300

Perego P, Giarola M, Righetti SC, Supino R, Caserini C, Delia D, Pierotti MA, Miyashita T, Reed JC and Zunino F (1996) Association between cisplatin resistance and mutation of p53 gene and reduced bax expression in ovarian carcinoma cell systems. *Cancer Res* **56**: 556–562

Perego P, Righetti SC, Supino R, Delia D, Caserini C, Carenini N, Bedogne' B, Broome E, Krajewski S, Reed JC and Zunino F (1997) Role of apoptosis and apoptosis-related proteins in the cisplatin-resistant phenotype of human tumor cell lines. *Apoptosis* **2**: 540–548

Perego P, Caserini C, Gatti L, Carenini N, Romanelli S, Supino R, Colangelo D, Viano I, Leone R, Spinelli S, Pezzoni G, Manzotti C, Farrell N and Zunino F (1999) A novel trinuclear platinum complex overcomes cisplatin resistance in an osteosarcoma cell system. *Mol. Pharmacol* **5**: 528–534

Righetti SC, Della Torre G, Pilotti S, Menard S, Ottone F, Colnaghi MI, Pierotti MA, Lavarino C, Cornarotti M, Oriana S, Bohm S, Bresciani GL, Spatti G and Zunino F (1996) A comparative study of p53 gene mutations, protein accumulation, and response to cisplatin-based chemotherapy in advanced ovarian carcinoma. *Cancer Res* **56**: 689–693

Rolley N, Butcher S and Milner J (1995) Specific DNA binding by different classes of human p53 mutants. *Oncogene* **11**: 763–770

Supino R, Righetti SC, Magnani I, Bottiroli G and Prosperi E (1996) Modulation and cell cycle distribution of mutant p53 protein in a human small-cell lung carcinoma cell line after exposure to cytotoxic agents. *Cell Pharmacol* **3**: 253–260

Vogelstein B and Kinzler KW (1994) X-rays strike p53 again. *Nature* **370**: 174–175

Wang XW, Yeh H, Schaeffer L, Roy R, Moncollin V, Egly J-M, Wang Z, Freidberg EC, Evans MK, Taff BG, Bohr VA, Weeda G, Hoeijmakers JHJ, Forrester K and Harris CC (1995) p53 modulation of TFIIH-associated nucleotide excision repair activity. *Nat Genet* **10**: 188–195

Workman P, Balmain A, Hickman JA, McNally NJ, Mitchison NA, Pierrepoint CG, Raymond R, Rowlatt C, Stephens TC and Wallace J (1988) UKCCCR guidelines for the welfare of animals in experimental neoplasia. *Br J Cancer* **58**: 109–113

Wu P, Kharatishvili M and Farrell N (1996) A circular dichroism study of ethidium bromide binding to Z-DNA induced by dinuclear platinum complexes. *J Inorg Biochem* **63**: 9–16

Zunino F, Perego P, Pilotti S, Pratesi G, Supino R and Arcamone F (1997) Role of apoptotic response in cellular resistance to cytotoxic agents. *Pharmacol Ther* **76**: 177–185

© 1999 Cancer Research Campaign

## References

[PDF_00672] Brachmann R. K., Vidal M., Boeke J. D. (1996). Dominant-negative p53 mutations selected in yeast hit cancer hot spots.. Proc Natl Acad Sci U S A.

[PDF_00675] Caserini C., Pratesi G., Tortoreto M., Bedogné B., Carenini N., Supino R., Perego P., Righetti S. C., Zunino F. (1997). Apoptosis as a determinant of tumor sensitivity to topotecan in human ovarian tumors: preclinical in vitro/in vivo studies.. Clin Cancer Res.

[PDF_00679] Chu G. (1994). Cellular responses to cisplatin. The roles of DNA-binding proteins and DNA repair.. J Biol Chem.

[PDF_00680] Crul M., Schellens J. H., Beijnen J. H., Maliepaard M. (1997). Cisplatin resistance and DNA repair.. Cancer Treat Rev.

[PDF_00682] Eastman A. (1987). The formation, isolation and characterization of DNA adducts produced by anticancer platinum complexes.. Pharmacol Ther.

[PDF_00692] Farrell N., Appleton T. G., Qu Y., Roberts J. D., Fontes A. P., Skov K. A., Wu P., Zou Y. (1995). Effects of geometric isomerism and ligand substitution in bifunctional dinuclear platinum complexes on binding properties and conformational changes in DNA.. Biochemistry.

[PDF_00684] Farrell N. (1993). Nonclassical platinum antitumor agents: perspectives for design and development of new drugs complementary to cisplatin.. Cancer Invest.

[PDF_00700] Fink D., Nebel S., Aebi S., Zheng H., Cenni B., Nehmé A., Christen R. D., Howell S. B. (1996). The role of DNA mismatch repair in platinum drug resistance.. Cancer Res.

[PDF_00703] Harris C. C. (1996). Structure and function of the p53 tumor suppressor gene: clues for rational cancer therapeutic strategies.. J Natl Cancer Inst.

[PDF_00705] Hecker D., Page G., Lohrum M., Weiland S., Scheidtmann K. H. (1996). Complex regulation of the DNA-binding activity of p53 by phosphorylation: differential effects of individual phosphorylation sites on the interaction with different binding motifs.. Oncogene.

[PDF_00709] Hickman J. A. (1996). Apoptosis and chemotherapy resistance.. Eur J Cancer.

[PDF_00711] Johnson A., Qu Y., Van Houten B., Farrell N. (1992). B----Z DNA conformational changes induced by a family of dinuclear bis(platinum) complexes.. Nucleic Acids Res.

[PDF_00714] Kastan M. B., Canman C. E., Leonard C. J. (1995). P53, cell cycle control and apoptosis: implications for cancer.. Cancer Metastasis Rev.

[PDF_00716] Kelland L. R. (1994). The molecular basis of cisplatin sensitivity/resistance.. Eur J Cancer.

[PDF_00718] Lowe S. W., Bodis S., McClatchey A., Remington L., Ruley H. E., Fisher D. E., Housman D. E., Jacks T. (1994). p53 status and the efficacy of cancer therapy in vivo.. Science.

[PDF_00721] O'Connor P. M., Jackman J., Bae I., Myers T. G., Fan S., Mutoh M., Scudiero D. A., Monks A., Sausville E. A., Weinstein J. N. (1997). Characterization of the p53 tumor suppressor pathway in cell lines of the National Cancer Institute anticancer drug screen and correlations with the growth-inhibitory potency of 123 anticancer agents.. Cancer Res.

[PDF_00735] Perego P., Caserini C., Gatti L., Carenini N., Romanelli S., Supino R., Colangelo D., Viano I., Leone R., Spinelli S. (1999). A novel trinuclear platinum complex overcomes cisplatin resistance in an osteosarcoma cell system.. Mol Pharmacol.

[PDF_00727] Perego P., Giarola M., Righetti S. C., Supino R., Caserini C., Delia D., Pierotti M. A., Miyashita T., Reed J. C., Zunino F. (1996). Association between cisplatin resistance and mutation of p53 gene and reduced bax expression in ovarian carcinoma cell systems.. Cancer Res.

[PDF_00732] Perego P., Righetti S. C., Supino R., Delia D., Caserini C., Carenini N., Bedogné B., Broome E., Krajewski S., Reed J. C. (1997). Role of apoptosis and apoptosis-related proteins in the cisplatin-resistant phenotype of human tumor cell lines.. Apoptosis.

[PDF_00739] Righetti S. C., Della Torre G., Pilotti S., Ménard S., Ottone F., Colnaghi M. I., Pierotti M. A., Lavarino C., Cornarotti M., Oriana S. (1996). A comparative study of p53 gene mutations, protein accumulation, and response to cisplatin-based chemotherapy in advanced ovarian carcinoma.. Cancer Res.

[PDF_00744] Rolley N., Butcher S., Milner J. (1995). Specific DNA binding by different classes of human p53 mutants.. Oncogene.

[PDF_00755] (1988). UKCCCR guidelines for the welfare of animals in experimental neoplasia.. Br J Cancer.

[PDF_00750] Vogelstein B., Kinzler K. W. (1994). Tumour-suppressor genes. X-rays strike p53 again.. Nature.

[PDF_00751] Wang X. W., Yeh H., Schaeffer L., Roy R., Moncollin V., Egly J. M., Wang Z., Freidberg E. C., Evans M. K., Taffe B. G. (1995). p53 modulation of TFIIH-associated nucleotide excision repair activity.. Nat Genet.

[PDF_00759] Wu P. K., Kharatishvili M., Qu Y., Farrell N. (1996). A circular dichroism study of ethidium bromide binding to Z-DNA induced by dinuclear platinum complexes.. J Inorg Biochem.

[PDF_00762] Zunino F., Perego P., Pilotti S., Pratesi G., Supino R., Arcamone F. (1997). Role of apoptotic response in cellular resistance to cytotoxic agents.. Pharmacol Ther.

